# Supplementary material for: Metabolic Engineering for Efficient Production of Z,Z-Farnesol in E. coli
Source: Microorganisms. 2023 Jun 15;11(6):1583. doi: 10.3390/microorganisms11061583 (PMC10303002; doi:10.3390/microorganisms11061583)
Supplement: Supplementary file 1 [file microorganisms-11-01583-s001.zip › microorganisms-2406861-supplementary.pdf]

Supplementary Materials

Metabolic Engineering for Efficient Production of Z,Z-Farnesol in *E. coli*

Mengyang Lei [1,2], Zetian Qiu [1,2], Leilei Guan [1,2], Zheng Xiang [3,*] and Guang-Rong Zhao [1,2,*]

1 Frontiers Science Center for Synthetic Biology, Key Laboratory of Systems Bioengineering (Ministry of Education), School of Chemical Engineering and Technology, Tianjin University, Yaguan Road 135, Jinnan District, Tianjin 300350, China; mylei@tju.edu.cn (M.L.); ztqiu@tju.edu.cn (Z.Q.); guan@tju.edu.cn (L.G.)

2 Georgia Tech Shenzhen Institute, Tianjin University, Dashi Road 1, Nanshan District, Shenzhen 518055, China

3 State Key Laboratory of Chemical Oncogenomics, School of Chemical Biology and Biotechnology, Peking University Shenzhen Graduate School, Shenzhen 518055, China

* Correspondence: xiangzheng@pkusz.edu.cn (Z.X.); grzhao@tju.edu.cn (G.-R.Z.)

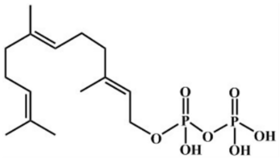

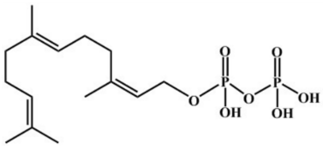

E,E-FPP **Z,E-FPP**

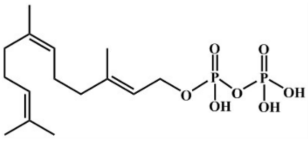

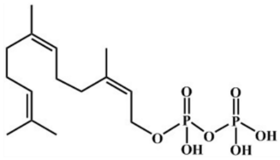

E,Z-FPP **Z,Z-FPP**

Figure S1. Chemical Structure of Four Isomers of Farnesyl Diphosphate. (E,Z-FPP synthase has not been isolated so far)

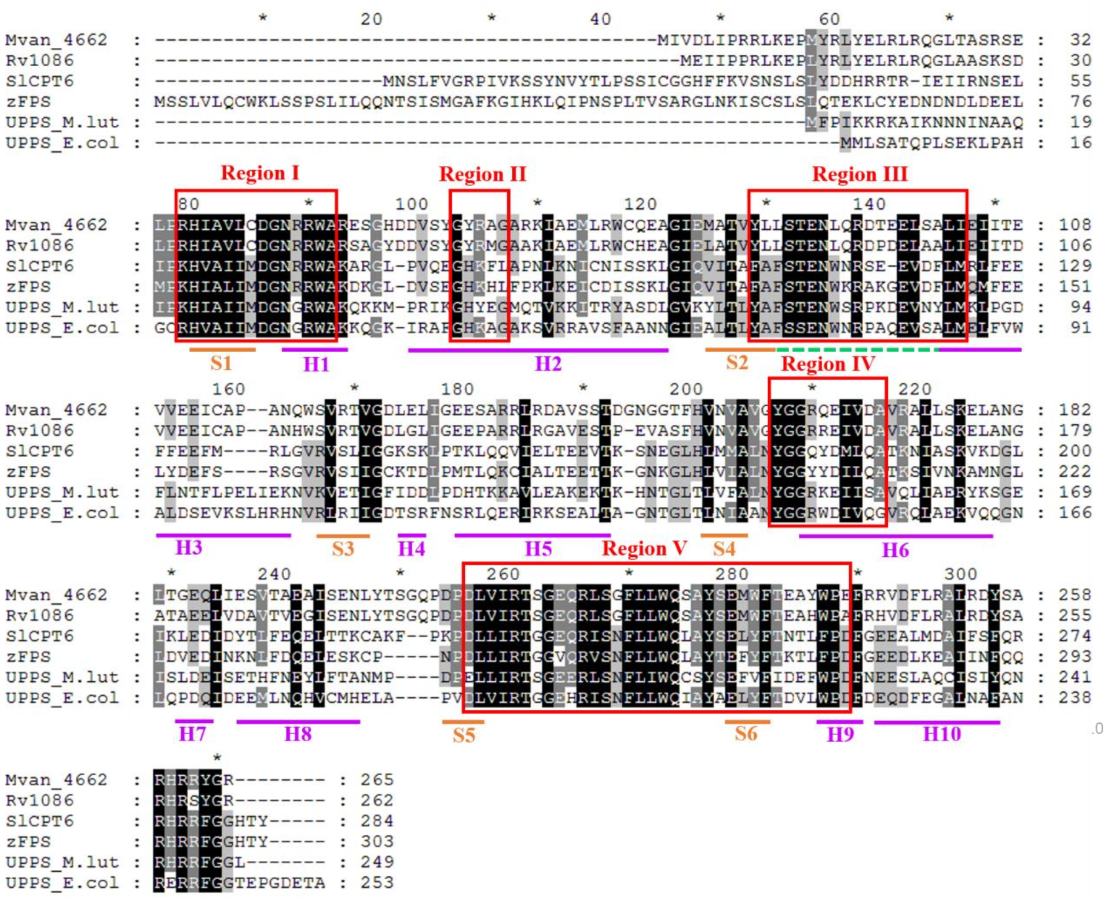

Figure S2. Multiple alignment of amino acid sequences of six *cis*-prenyltransferases: Mvan_4662, Rv1086, SlCPT6, zFPS, UPPS from *M. luteus* (BAA31993), UPPS from *E.coli* (ASI52648.1). ‘*’ represents the symbol of marker line. Orange underlines indicate β-sheets and Purple underlines indicate α-helices. Residues in conserved regions I to V has been boxed and flexible domain in region III has been drawn with a green dotted line.

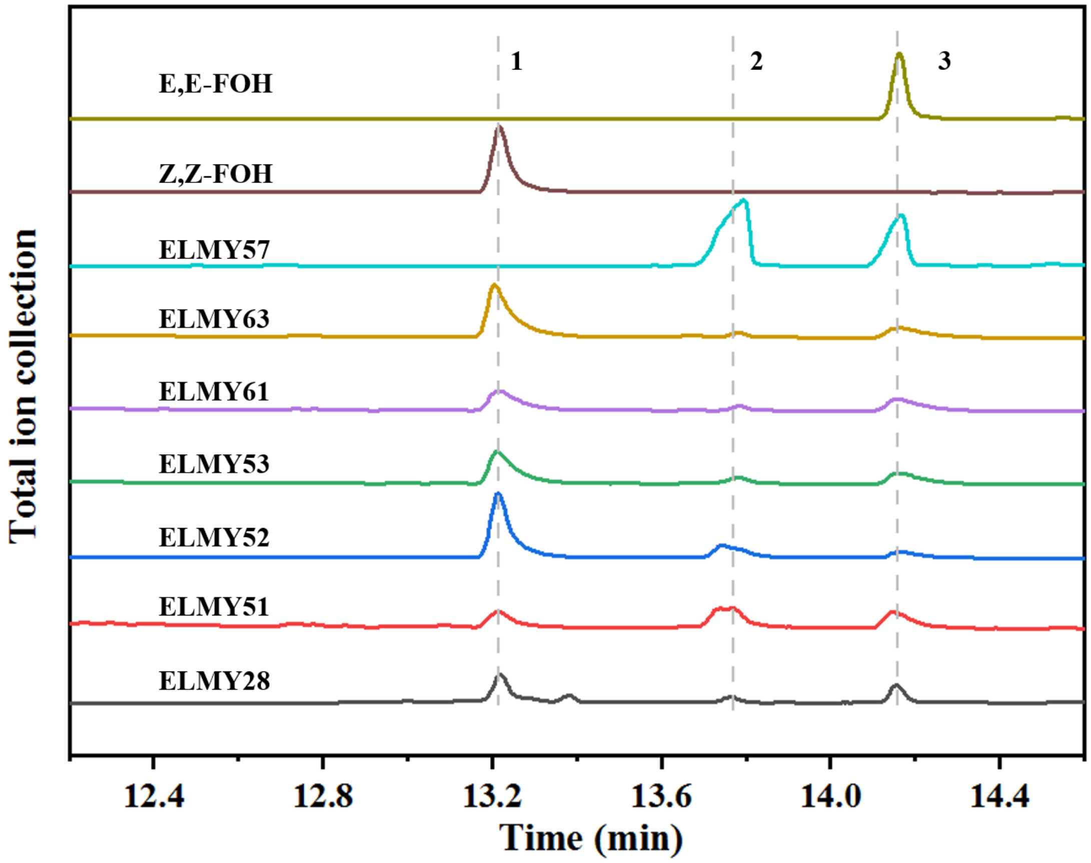

Figure S3. GC chromatogram of cultures by engineered strains and FOH isomers standard. Peak 1 represents Z,Z-FOH, peak 2 represents Z,E-FOH, peak 3 represents E,E-FOH.

Table S1. Primers used in this study

Primer	sequence
SltNPPS-F	ATAAGGAGATATACCATGTCTGCTAGAGGTTTGAACA
SltNPPS-R	GCGGCCGCAAGCTTTTAGTAAGTGTGACCACCGAATC
SltNPPS-Vec-F	ACACTTACTAAAAGCTTGCGGCCG
SltNPPS-Vec-R	TAGCAGACATGGTATATCTCCTTATTAAAGTTAAACAAAATTATTT
PstIDS3-F	AGATATACATATGACCCTGGCGAGC
PstIDS3-R	AGACTCGAGGTTAGCAAACGCTCTTGCTG
PstIDS3-Vec-F	AGCAAGAGCGTTTGCTAACCTCGAGTCTGGTAAAGAAAC
PstIDS3-Vec-R	AAAGCTCGCCAGGGTCATATGTATATCTCCTTCTTATACTTAACTAATATACTAAG
SltCPT6-F	AGATATACATATGAGCAGCTACAACG
SltCPT6-R	AGACTCGAGGTTAATAGGTGTGGCCACC
SltCPT6-Vec-F	GGTGGCCACACCTATTAACCTCGAGTCTGGTAAAGAAAC
SltCPT6-Vec-R	CACGTTGTAGCTGCTCATATGTATATCTCCTTCTTATACTTAACTAATATACTAAG
tZFPS-F	AGATATACATATGGCGCGTGGTCTGAAC
tZFPS-R	AGACTCGAGGTTAATAGGTGTGGCCACCAAAACGAC
tZFPS-Vec-F	GGTGGCCACACCTATTAACCTCGAGTCTGGTAAAGAAAC
tZFPS-Vec-R	GTTCAGACCACGCGCCATATGTATATCTCCTTCTTATACTTAACTAATATACTAAG
Mvan_4662-F	GAAGGAGATATACATATGATCGTTGACCTGATTCC
Mvan_4662-R	TTACCAGACTCGAGGTTAACGTCCATAACGACGATGAC
Mvan_4662-Vec-F	TGGACGTTAACCTCGAGTCTGGTAAAGAAAC
Mvan_4662-Vec-R	CAACGATCATATGTATATCTCCTTCTTATACTTAACTAATATACTAAG
Rv1086-F	GAAGGAGATATACATATGGAGATCATTCCGCCG
Rv1086-R	AGACTCGAGGTTAACGACCATAGCTACGGTG
Rv1086-Vec-F	AGCTATGGTCGTTAACCTCGAGTCTGGTAAAGAAAC
Rv1086-Vec-R	TGATCTCCATATGTATATCTCCTTCTTATACTTAACTAATATACT
rdgB-F	GAGATATACCATGCAAAAAGTTGTCCTCGC

rdgB-R	GACTTAAGCATTAACCATTACGTAAAGCGTCCA
rdgB-Vec-F	GCTTTACGTAATGGTTAATGCTTAAGTCGAACAGAAAGTAATC
rdgB-Vec-R	GAGGACAACTTTTTGCATGGTATATCTCCTTATTAAAGTTAAACAAAATTATTTC
phoA-F	GAGATATACCATGCCTGTTCTGGAAAACC
phoA-R	GACTTAAGCATTATTTCAGCCCCAGAGCG
phoA-Vec-F	GCTCTGGGGCTGAAATAATGCTTAAGTCGAACAGAAAGTAATC
phoA-Vec-R	GTTTTCCAGAACAGGCATGGTATATCTCCTTATTAAAGTTAAACAAAATTATTTC
ppa-F	GAGATATACCATGAGCTTACTCAACGTCCC
ppa-R	GACTTAAGCATTATTTATTCTTTGCGCGCTCG
ppa-Vec-F	CGCGCAAAGAATAAATAATGCTTAAGTCGAACAGAAAGTAATC
ppa-Vec-R	GACGTTGAGTAAGCTCATGGTATATCTCCTTATTAAAGTTAAACAAAATTATTTC
cdh-F	GAGATATACCATGAAAAAAGCGGGTCTTCTT
cdh-R	GACTTAAGCATTAACGCAAAATCTCACACTGAT
cdh-Vec-F	TGTGAGATTTTGCGTTAATGCTTAAGTCGAACAGAAAGTAATC
cdh-Vec-R	AAGACCCGCTTTTTTCATGGTATATCTCCTTATTAAAGTTAAACAAAATTATTTC
lpxH-F	GAGATATACCATGGCGACACTCTTTATTGC
lpxH-R	GACTTAAGCATTAAAACGGAAAATGAATCAGCTCA
lpxH-Vec-R	AATAAAGAGTGTCGCCATGGTATATCTCCTTATTAAAGTTAAACAAAATTATTTC
lpxH-Vec-F	ATTCATTTTCCGTTTTAATGCTTAAGTCGAACAGAAAGTAATC
mutT-F	GAGATATACCATGAAAAAGCTGCAAATTGCG
mutT-R	GACTTAAGCATTACAGACGTTTAAGCTTCGC
mutT-Vec-F	AAGCTTAAACGTCTGTAATGCTTAAGTCGAACAGAAAGTAATC
mutT-Vec-R	AATTTGCAGCTTTTTCATGGTATATCTCCTTATTAAAGTTAAACAAAATTATTTC
nudc-F	GAGATATACCATGGATCGTATAATTGAAAAATTAGATCAC
nudC-R	GACTTAAGCATTACTCATACTCTGCCCGACA
nudC-Vec-F	CGGGCAGAGTATGAGTAATGCTTAAGTCGAACAGAAAGTAATC
nudC-Vec-R	TTCAATTATACGATCCATGGTATATCTCCTTATTAAAGTTAAACAAAATTATTTC
nudF-F	GAGATATACCATGCTTAAGCCAGACAACC

nudF-R	GACTTAAGCATTATGCCCACTCATTTTTAACGC
nudF-Vec-F	AAAAATGAGTGGGCATAATGCTTAAGTCGAACAGAAAGTAATC
nudF-Vec-R	GTTGTCTGGCTTAAGCATGGTATATCTCCTTATTAAAGTTAAACAAAATTATTTC
nudj-F	GAGATATACCATGTTTAAACCGCACGTTACC
nudj-R	GACTTAAGCATTAGATGACACCCTTTGTAAAAGGC
nudj-Vec-F	ACAAAGGGTGTCATCTAATGCTTAAGTCGAACAGAAAGTAATCG
nudj-Vec-R	AACGTGCGGTTTAAACATGGTATATCTCCTTATTAAAGTTAAACAAAATTAT
C41A-F	CGTTCTGGCAGATGGAAATCGTCGTTGGGCAC
C41A-R	GATTTCCATCTGCCAGAACGGCAATGTGGCGC
C41F-F	CGTTCTGTTTGATGGAAATCGTCGTTGGGCAC
C41F-R	GATTTCCATCAAACAGAACGGCAATGTGGCGC
C41L-F	CGTTCTGCTGGATGGAAATCGTCGTTGGGCAC
C41L-R	GATTTCCATCCAGCAGAACGGCAATGTGGCGC
C41M-F	CGTTCTGATGGATGGAAATCGTCGTTGGGCAC
C41M-R	GATTTCCATCCATCAGAACGGCAATGTGGCGC
C41S-F	CGTTCTGAGCGATGGAAATCGTCGTTGGGCAC
C41S-R	GATTTCCATCGCTCAGAACGGCAATGTGGCGC
C41W-F	CGTTCTGTGGGATGGAAATCGTCGTTGGGCAC
C41W-R	GATTTCCATCCCACAGAACGGCAATGTGGCGC
C41Y-F	CGTTCTGTATGATGGAAATCGTCGTTGGGCAC
C41Y-R	GATTTCCATCATACAGAACGGCAATGTGGCGC
Y85A-F	GCAACCGTTGCACTGCTGAGCACAGAAAATCTGCAG
Y85A-R	GCTCAGCAGTGCAACGGTTGCCATTTCAATACCTGC
Y85F-F	GCAACCGTTTTTCTGCTGAGCACAGAAAATCTGCAG
Y85F-R	GCTCAGCAGAAAAACGGTTGCCATTTCAATACCTGC
Y85L-F	GCAACCGTTCTGCTGCTGAGCACAGAAAATCTGCAG
Y85L-R	GCTCAGCAGCAGAACGGTTGCCATTTCAATACCTGC
Y85S-F	GCAACCGTTAGCCTGCTGAGCACAGAAAATCTGCAG
Y85S-R	GCTCAGCAGGCTAACGGTTGCCATTTCAATACCTGC

Y85W-F	GCAACCGTTTGGCTGCTGAGCACAGAAAATCTGCAG
Y85W-R	GCTCAGCAGCCAAACGGTTGCCATTTCAATACCTGC
D95A-F	TGCAGCGTGCAACAGAAGAACTGAGCGCACTGA
D95A-R	GTTCTTCTGTTGCACGCTGCAGATTTTCTGTGCT
D95P-F	TGCAGCGTCCAACAGAAGAACTGAGCGCACTGA
D95P-R	GTTCTTCTGTTGGACGCTGCAGATTTTCTGTGCT
D95S-F	TGCAGCGTAGCACAGAAGAACTGAGCGCACTGA
D95S-R	GTTCTTCTGTGCTACGCTGCAGATTTTCTGTGCT
E97D-F	GTGATACAGATGAACTGAGCGCACTGATTGAAATCA
E97D-R	CGCTCAGTTCATCTGTATCACGCTGCAGATTTTCTGTGC
E97G-F	GTGATACAGGTGAACTGAGCGCACTGATTGAAATCA
E97G-R	CGCTCAGTTCACCTGTATCACGCTGCAGATTTTCTGTGC
E97Q-F	GTGATACACAGGAACTGAGCGCACTGATTGAAATCA
E97Q-R	CGCTCAGTTCCTGTGTATCACGCTGCAGATTTTCTGTGC
L92A-F	CAGAAAATGCACAGCGTGATACAGAAGAACTGAGC
L92A-R	TATCACGCTGTGCATTTTCTGTGCTCAGCAGATAAACGG
L92F-F	CAGAAAATTTTCAGCGTGATACAGAAGAACTGAGC
L92F-R	TATCACGCTGAAAATTTTCTGTGCTCAGCAGATAAACGG
L92S-F	CAGAAAATAGCCAGCGTGATACAGAAGAACTGAGC
L92S-R	TATCACGCTGGCTATTTTCTGTGCTCAGCAGATAAACGG
L92W-F	CAGAAAATTGGCAGCGTGATACAGAAGAACTGAGC
L92W-R	TATCACGCTGCCAATTTTCTGTGCTCAGCAGATAAACGG
L99V-F	CAGAAGAAGTTAGCGCACTGATTGAAATCATTACCG
L99V-R	TCAGTGCGCTAACTTCTTCTGTATCACGCTGCAGATTTTCT
Q93A-F	AAATCTGGCACGTGATACAGAAGAACTGAGCGC
Q93A-R	TCTGTATCACGTGCCAGATTTTCTGTGCTCAGCAGATAAACG
Q93G-F	AAATCTGGGTCGTGATACAGAAGAACTGAGCGC
Q93G-R	TCTGTATCACGACCCAGATTTTCTGTGCTCAGCAGATAAACG
Q93K-F	AAAATCTGAAACGTGATACAGAAGAACTGAGCGC
Q93K-R	CTGTATCACGTTTCAGATTTTCTGTGCTCAGCAGATAAACG

Q93S-F	AAAATCTGAGCCGTGATACAGAAGAACTGAGCGC
Q93S-R	CTGTATCACGGCTCAGATTTTCTGTGCTCAGCAGATAAACG
Q93N-F	AAAATCTGAATCGTGATACAGAAGAACTGAGCGC
Q93N-R	CTGTATCACGATTCAGATTTTCTGTGCTCAGCAGATAAACG

Table S2. Codon-optimized sequences of *mvan4662*, *rv1086* and truncated *PsIDS3*, *SlCPT6*, *ShzFPS* and *TcNudix1* in this study.

Gene	Encoding sequence
mvan4662	ATGATCGTTGACCTGATTCCGCGCCGTCTGAAAGAGCCGA<br>TGTACCGTCTGTATGAACTGCGTCTGCGCCAGGGCCTGACC<br>GCAAGTCGTTCCGAGCTGCCGCGCCACATTGCCGTTCTGTG<br>TGATGGAAATCGTCGTTGGGCACGTGAAAGCGGTCACGAT<br>GATGTTAGCTATGGTTATCGTGCAGGAGCACGCAAAATTG<br>CAGAAATGCTGCGTTGGTGTCAGGAAGCAGGTATTGAAAT<br>GGCAACCGTTTATCTGCTGAGCACAGAAAATCTGCAGCGT<br>GATACAGAAGAACTGAGCGCACTGATTGAAATCATTACCG<br>AGGTTGTTGAAGAAATTTGTGCACCAGCAAACCAGTGGAG<br>TGTTCGTACCGTTGGTGATCTGGAACTGATTGGAGAGGAA<br>AGCGCACGTCGTCTGCGTGATGCAGTGAGCAGCACCGATG<br>GGAATGGAGGGACCTTTCATGTGAATGTGGCAGTGGGTTA<br>TGGTGGTCGTCAGGAAATTGTTGATGCGGTTCGTGCACTGC<br>TGAGTAAAGAACTGGCGAATGGTCTGACAGGCGAACAGCT<br>GATTGAGTCAGTTACCGCAGAGGCAATTAGTGAAAATCTG<br>TATACCAGCGGGCAGCCGGATCCTGATCTGGTTATTCGTAC<br>AAGCGGAGAACAACGCCTGAGCGGCTTTCTGCTGTGGCAG<br>AGCGCATATAGCGAAATGTGGTTTACCGAAGCGTATTGGC<br>CGGAGTTTCGTCGTGTTGATTTTCTGCGTGCCCTGCGCGAT<br>TATAGCGCACGTCATCGTCGTTATGGACGTTAA
rv1086	ATGGAGATCATTCCGCCGCGTCTGAAAGAGCCGCTGTACC<br>GTCTGTATGAGCTGCGTCTGCGTCAAGGCCTGGCGGCGAG<br>CAAGAGCGACCTGCCGCGTCACATTGCGGTTCTGTGCGAT<br>GGTAACCGTCGTTGGGCGCGTAGCGCGGGTTACGACGATG<br>TGAGCTACGGCTATCGTATGGGTGCGGCGAAGATCGCGGA<br>GATGCTGCGTTGGTGCCACGAGGCGGGTATTGAACTGGCG<br>ACCGTGTATCTGCTGAGCACCGAAAACCTGCAGCGTGACC<br>CGGATGAGCTGGCGGCGCTGATCGAAATCATTACCGACGT<br>GGTTGAGGAAATTTGCGCGCCGGCGAACCACTGGAGCGTT<br>CGTACCGTGGGTGATCTGGGCCTGATTGGTGAAGAACCGG<br>CGCGTCGTCTGCGTGGCGCGGTTGAGAGCACCCCGGAAGT

GGCGAGCTTCCACGTGAACGTTGCGGTGGGTTATGGTGGC
CGTCGTGAGATTGTTGACGCGGTGCGTGCGCTGCTGAGCA
AGGAACTGGCGAACGGTGCGACCGCGGAGGAACTGGTTG
ATGCGGTTACCGTGGAGGGCATCAGCGAAAACCTGTACAC
CAGCGGTCAGCCGGACCCGGATCTGGTGATTCGTACCAGC
GGCGAACAACGTCTGAGCGGTTTCCTGCTGTGGCAGAGCG
CGTATAGCGAGATGTGGTTTACCGAAGCGCACTGGCCGGC
GTTCCGTCACGTTGACTTTCTGCGTGCGCTGCGTGATTACA
GCGCGCGTCACCGTAGCTATGGTCGTTAA

PstIDS3 ATGACCCTGGCGAGCTTTAGCGACGCGGATACCGAGGAGT
TCATGAGCCACTTTCCGACCATCGTGGAGGACCTGAGCAC
CGGTCTGAACCTGAAGGACATGAAACAGACCGTTGATCGT
TTCGCGAACTGCGTGCGTACCAACGTTCCGAACGGTAAGA
AAGTGCGTGGCCTGACCACCGTTATTACCTACAAGCTGCT
GGAGAAACCGGAAAACCTGACCCCGGACAACATCCGTCTG
GCGAACATTCTGGGCTGGTTTATCGAGCTGATTGAAGCGG
CGGTGGTTATCTTCGACGATGTGATGGACAACAGCTTTACC
CGTCGTGGTGGCCTGTGCTGGCACCGTCAGAAAAACGTGG
GTGTTCTGGCGATTGCGGATGGCCTGAGCCTGAACAGCGT
GGCGTACCTGCTGCTGGAGAAGCACTTCCGTAACCACCCG
CACTTTGTTGACATGTTCTATTTTAGCAAGAGCATCATGTT
CAAAACCACCCTGGGTCAGGCGGCGGACAGCACCCGTCAT
GCGCCGGAAACCATCAGCCTGGATAAGTTCAAGACCATCG
CGAAGTACAAGACCGGCTTCTATACCTTTTACTATAGCGTG
GGTCTGGGCATGTACCTGAGCCGTCAGTTCAACAAAGACG
TTCTGGAGAACCAAATTGCGCCGCTGCTGTTCGATATGGGT
GAATACTTCCAAATCAAGAACGACATCAACGATTGCTTTG
TGAAAGAGGACATCGCGGGTAAAACCGGCACCGATATTGA
GGAAAGCAAATGCACCTGGCTGGCGGTTAAGGCGCTGGAG
AAGGGTAACGAAGCGCAGAAGGCGCTGTTCCGTGAAAACT
ACGGCAAGGCGGACCCGAAAAGCGTGGAGAGCATCCGTA
ACCTGTACCGTGAACTGAAACTGCAAGAGGAATTTCACGA
GTATGAAACCAACTTCTTTAAGAGCCTGCGTAACCGTAGC
CTGAGCATCGAGAGCCCGTATAGCAACGTGACGATGGTGC

	TGATTGAGAAGCTGCAGCGTCAAGTGTACAAAACCGTTGA
	ATATAACAGCAAGAGCGTTTGCTAA
SltCPT6	ATGAGCAGCTACAACGTGTATACCCTGCCGAGCAGCATTT
	GCGGTGGCCACTTCTTTAAGGTTAGCAACAGCCTGAGCCT
	GTACGACGATCACCGTCGTACCCGTATCGAGATCATTCGT
	AACAGCGAACTGATTCCGAAACACGTGGCGATCATTATGG
	ACGGTAACCGTCGTTGGGCGAAGGCGCGTGGTCTGCCGGT
	TCAGGAAGGTCACAAATTCCTGGCGCCGAACCTGAAGAAC
	ATCTGCAACATTAGCAGCAAACTGGGCATCCAAGTGATTA
	CCGCGTTCGCGTTTAGCACCGAAAACTGGAACCGTAGCGA
	GGAAGTTGATTTCCTGATGCGTCTGTTTGAGGAGTTCTTCG
	AGGAATTTATGCGTCTGGGTGTGCGTGTTAGCCTGATCGGT
	GGCAAGAGCAAACTGCCGACCAAACTGCAGCAAGTGATTG
	AGCTGACCGAGGAAGTTACCAAGAGCAACGAAGGTCTGC
	ATCTGATGATGGCGCTGAACTACGGTGGCCAGTATGACAT
	GCTGCAAGCGACCAAAAACATCGCGAGCAAGGTGAAAGA
	TGGCCTGATCAAGCTGGAGGACATTGATTACACCCTGTTC
	GAGCAGGAACTGACCACCAAGTGCGCGAAATTTCCGAAGC
	CGGACCTGCTGATCCGTACCGGTGGCGAGCAGCGTATTAG
	CAACTTCCTGCTGTGGCAACTGGCGTACAGCGAACTGTATT
	TTACCAACACCCTGTTCCCGGACTTTGGCGAGGAAGCGCT
	GATGGATGCGATCTTCAGCTTTCAACGTCGTCACCGTCGTT
	TCGGTGGCCACACCTATTAA
ShtzFPS	ATGGCGCGTGGTCTGAACAAGATCAGCTGCAGCCTGAGCC
	TGCAAACCGAGAAACTGTGCTACGAAGACAACGATAACG
	ACCTGGATGAGGAACTGATGCCGAAACACATCGCGCTGAT
	TATGGACGGCAACCGTCGTTGGGCGAAGGACAAAGGTCTG
	GATGTGAGCGAGGGCCACAAGCACCTGTTCCCGAAGCTGA
	AAGAAATCTGCGATATTAGCAGCAAACTGGGTATCCAGGT
	TATTACCGCGTTCGCGTTTAGCACCGAGAACTGGAAGCGT
	GCGAAAGGCGAAGTTGACTTCCTGATGCAAATGTTTGAGG
	AACTGTACGATGAGTTTAGCCGTAGCGGTGTGCGTGTTAG
	CATCATTGGCTGCAAGACCGACCTGCCGATGACCCTGCAA
	AAATGCATCGCGCTGACCGAGGAAACCACCAAGGGTAAC
	AAAGGTCTGCATCTGGTGATTGCGCTGAACTATGGTGGCT

	ACTATGACATCCTGCAGGCGACCAAGAGCATTGTGAACAA<br>AGCGATGAACGGTCTGCTGGACGTTGAAGATATCAACAAG<br>AACCTGTTCGATCAAGAGCTGGAAAGCAAATGCCCGAACC<br>CGGACCTGCTGATTCGTACCGGTGGCGATCAGCGTGTTAG<br>CAACTTTCTGCTGTGGCAACTGGCGTACACCGAGTTCTATT<br>TTACCAAGACCCTGTTCCCGGACTTTGGCGAGGAAGACCT<br>GAAAGAAGCGATCATTAACTTCCAGCAACGTCACCGTCGT<br>TTTGGTGGCCACACCTATTAA
TctNudix1	ATGCAAAACAAGGAAAGAGCTTTCTCTCCAAACAGAAGTA<br>TCTTCCAAACTTCTGATACCAGATTGATTGATGTCGAAGCT<br>TCCTCCGTTGAAAATGCTGCTAAGTCCAGAGTTCCAGAAG<br>TTGGTGTTACTGTTTTCATCGTCAAGGATGACAAGATCTTG<br>TTAGGTAGAAGAGGTAGCACTTTGGTCGTCGGTAACTCCTT<br>CCACTTACCAGGTGGTCATTTGGAATACGGTGAATCCTTTG<br>AGGAATGTGCCACCCGTGAAGTCAAGGAAGAAACAGGTTT<br>GGACATTAACAACTTGAAGGTTGTCACCATTGTCAACCAC<br>GTTTTGGCTGATATTCACGCTGTCGTTGTCTACATGAGAGC<br>CAATTTATCTGACCCAAACCAAACTCCACAAACCATCGAA<br>CCTCAAAGATGTGAAGGTTGGGACTGGTACGACTTGGAAA<br>ACCTGCCAGAACCAATGTTCCAACCATTGAAAGAATTGTT<br>GCAAGCTGGTTCTTTCAACATTTTCACCACTCACTCTTAA

Table S3. Description of phosphatases used for Z,Z-FOH production in this study。

Names	Accessions	Descriptions
RdgB	NP_417429.1	dITP/XTP pyrophosphatase
PhoA	NP_414917.2	Periplasmic alkaline phosphatase
ScLPP1	NP_010791.3	lipid phosphate phosphatase
Ppa	NP_418647.1	Inorganic pyrophosphatase
Cdh	NP_418353.1	CDP-diacylglycerol pyrophosphatase
LpxH	NP_415057.1	UDP-2,3-diacylglucosamine hydrolase
MutT	NP_414641.1	8-Oxo-dGTP diphosphatase
NudC	YP_026280.1	NADH pyrophosphatase
NudF	NP_417506.1	ADP-ribose pyrophosphatase
Nudj	NP_415652.1	Nudix hydrolase
TctNudix1	MT12604.1	Truncated chloroplast nudix1
PgpB	NP_415794.1	Phosphatidylglycerophosphatase B
YbgG	NP_415362.1	Undecaprenyl (C55) pyrophosphate
